# Detection and apparent survival of PIT‐tagged stream fish in winter

**DOI:** 10.1002/ece3.2061

**Published:** 2016-03-14

**Authors:** Christine Weber, Hannes Scheuber, Christer Nilsson, Knut T. Alfredsen

**Affiliations:** ^1^ Landscape Ecology GroupDepartment of Ecology and Environmental ScienceUmeå UniversitySE‐901 87UmeåSweden; ^2^ Tuefteln.chCH‐3302MoosseedorfSwitzerland; ^3^ Department of Hydraulic and Environmental EngineeringNorwegian University of Science and TechnologyNO‐7491TrondheimNorway

**Keywords:** Brown trout, European sculpin, ice, Sweden, tracking

## Abstract

Environmental fluctuations exert strong control on behavior, survival, and fitness of stream biota. Technical improvements increasingly allow for tracking the response of large numbers of individuals to environmental fluctuations, for instance, by remote detection of animals equipped with PIT (passive integrated transponder) tags. PIT tags were implanted into 393 juvenile and adult brown trout *Salmo trutta* L. and European sculpin *Cottus gobio* L. in a boreal stream subjected to considerable ice formation. With weekly trackings over 6 months, we quantified apparent survival and detection probability in relation to biological, environmental, and methodological factors. Individuals with a higher physical condition in autumn showed a higher apparent survival; this pattern was consistent across all species and age classes. Detection probability decreased with increasing thickness of the surface ice layer; this effect was most pronounced for juvenile trout and benthic‐living sculpin, both tagged with smaller‐sized tags. Detection probability was reduced in structurally complex habitats. Our study demonstrates that apparent survival and particularly detection probability may show pronounced spatiotemporal variation. In order to compare results from different sampling occasions and sites, a good knowledge of the study site and of the regulating factors is crucial.

## Introduction

River ecosystems are highly dynamic, with environmental conditions undergoing more frequent and more rapid spatiotemporal fluctuations than in terrestrial, marine, or lentic habitats (Power 2001). Environmental fluctuations shape riverine habitats and exert strong control on behavior, survival, and fitness of stream biota (Resh et al. 1988). In high‐latitude and high‐altitude rivers, environmental fluctuations follow a pronounced seasonal pattern. During the winter, snow, low temperatures, and various types of river ice can fundamentally alter hydraulics, discharge, or gaseous exchange (Prowse 2001). Winter is generally regarded as a bottleneck for stream biota (Heggenes et al. 1993), that is, as a period of increased susceptibility and mortality (Power et al. 1993). In cold water, ectothermic animals such as fish have reduced swimming abilities (Brown et al. 2011), their energy reserves continuously deplete, and their physical condition declines (Cunjak 1996). However, the winter season has been understudied, among others due to methodological difficulties under harsh environmental conditions (Huusko et al. 2007; Weber et al. 2013).

Over the past years, PIT (passive integrated transponder) technology has been improved and increasingly used in high‐altitude and latitude streams to explore how environmental fluctuations and particularly ice formation affect winter habitat use, activity, and physical condition in fish and how these factors influence demographic processes such as reproduction, mortality, immigration, and emigration (Roussel et al. 2004; Stickler et al. 2008; Linnansaari and Cunjak 2010). PIT tags differ from active location devices such as radio‐transmitters by being dormant until powered by an external reader. The detection range, that is, the maximum distance from the antenna at which a tag is detected (Linnansaari et al. 2007), depends on methodological and environmental factors such as tag size, diameter of the antenna, or electromagnetic disturbance. The detection range has been expanded to the decimeter to meter scale over the past years (Roussel et al. 2000; Gibbons and Andrews 2004; Linnansaari and Cunjak 2007; Palm et al. 2009).

Many winter tracking studies observed variable return rates, that is, the proportion of detected tagged individuals to the total number of tagged individuals differed between tracking occasions. For instance, Palm et al. (2009) detected 48% of the tagged juvenile brown trout in the autumn tracking, while 29% and 13% were found in follow‐up surveys in early and late winter, respectively. The return rate is the product of two probabilities (Cooch and White 2011) – the probability of surviving and remaining in or returning to the study area (“apparent survival”) and the detection probability. An isolated comparison of return rates can be misleading as similar values may mask differences in the underlying mechanisms (Horton and Letcher 2008) driven by methodological, environmental, or biological factors. For instance, apparent survival can vary with an individual's size and social status (territories; Crespin et al. 2008). Detection probability may be biased by too small of a study area (study design; Horton and Letcher 2008). A thorough understanding of factors that influence detection probability and apparent survival is therefore required for a sound interpretation of data.

We quantified apparent survival and detection probability of 393 PIT‐tagged brown trout (*Salmo trutta* L.) and European sculpin (*Cottus gobio* L) in a boreal stream over winter, by accounting for the influence of biological, environmental, and methodological factors. Our first objective was to determine to what degree apparent survival is affected by individual characteristics of fish such as physical condition in autumn or habitat use. Our second objective was to quantify detection probability under temporally dynamic environmental conditions such as the formation of different types of river ice. Third, we applied two different tag sizes which allowed us to account for the effect of tag size on detection probability, also by considering interactions with biological and environmental factors.

We hypothesized that detection probability would decrease with increasing thickness of the surface ice layer, with the lowest values observed for the individuals tagged with 12‐mm tags (sculpin and juvenile brown trout), and in deep, structurally complex habitats. We expected apparent survival over winter to be positively correlated with physical condition of fish in autumn.

## Materials and Methods

### Study reach characteristics and habitat mapping

The study was performed in a 550‐m long reach of Smörbäcken, a third‐order tributary to the Ume River in Västerbotten County, Sweden (63°56′ N; 20°02′ E). In this boreal environment, winter lasts from early November to early April (Swedish Meteorological and Hydrological Institute, SMHI), and rivers are subject to pronounced formation of ice. Their flow regime is snowmelt‐driven, with the highest water levels in May–June and the lowest in late winter. The Smörbäcken catchment is characterized by crystalline geology (Precambrian gneiss) and mixed boreal forest. The study reach has a near‐natural morphology.

In autumn 2010, we generated a detailed 3D map of the study reach by means of a total station (Geodolite 506 total station, Trimble, Sunnyvale, CA). On average, one cross‐sectional profile consisting five evenly distributed points was set every 2 m along the river course. In addition to the topographical measurements, water depth was determined at each point. Furthermore, we collected the positions of 221 reference trees for fish tracking (see below), distinctly marked with a code of plastic tape. Field data were processed in ArcMap 9.3.1 (ESRI Inc., Redlands, CA).

### Ice thickness

Average ice thickness *h*_*i*_ [m] across the study reach was approximated for weekly intervals following the Stefan equation approach (Ashton 1986) by accounting for reduced growth of ice when covered by snow (Lundberg and Feiccabrino 2009)

$$h_{i}={\left(\frac{2k_{s/i}}{\rho L}\right)}^{1/2}S^{1/2}$$

where *k*_*s/i*_ = thermal conductivity of the ice–snow layer [W m^−1^ K^−1^], *ρ *= density of the ice [kg m^−3^], *L *= latent heat of fusion of ice [J g^−1^], and *S =* number of degree‐days of freezing [°C day).

*ρ* was set to 1000 g/m^3^ (Dingman 2015), *L* was set to 333.4 J/g (Dingman 2015), and *S* was based on air temperature measurements at the study site.

*k*_*s/i*_ was calculated with the formula by Lundberg and Feiccabrino (2009)

$$k_{s/i}=\frac{h_{i}+h_{s}}{\frac{h_{i}}{k_{i}}+\frac{h_{s}}{k_{s}}}$$

where *k*_*i*_ = thermal conductivity of the ice layer [W m^−1^ K^−1^], *k*_*s*_ = thermal conductivity of the snow layer [W m^−1^ K^−1^], *h*_*s*_ = thickness of the snow layer [m].

Removal of excessive snow (see below) was accounted for by resetting thickness of the snow layer to 3 cm in *h*_*i*_ calculation after each of the three shoveling occasions. Calculated values of average ice thickness were compared with in situ measurements made at the end of January 2011 by drilling the ice at 121 positions distributed along the study reach.

### Fish assemblage and electrofishing

The study reach harbors high densities of European sculpin (20 year mean = 77 individuals 100 m^−1^; SD = 29 individuals 100 m^−1^) and brown trout (130 individuals 100 m^−1^; 73 individuals 100 m^−1^) with various age classes present. There were occasional occurrences of European perch *Perca fluviatilis* L., European brook lamprey *Lampetra planeri* Bloch, river lamprey *Lampetra fluviatilis* L., and northern pike *Esox lucius* L. (Swedish Electrofishing Register, SERS).

We divided the study reach into 11 subreaches of similar length (mean = 49.9 m; SD = 6.4 m), based on natural breaks in meso‐habitats (mapping according to Borsányi et al. 2004). The subreaches were quantitatively sampled by electrofishing (blocknets, three runs; White et al. 1982) in October 2010 and in May 2011 using a portable electroshocker (Lugab, Luleå, Sweden; 0.6 kW, 800 V; cDC). Captured fish were handled in accordance with a standardized procedure including controlled conditioning and anesthesia with MS 222 (Tricaine methanesulfonate, Sigma‐Aldrich, Buchs, Switzerland; 0.5 g diluted in 10 L water). Fish species, total length (±1 mm), weight (±1 g), and type of any anomalies were recorded.

### Fish tagging

The tagging procedure had been successfully tested in the laboratory with wild brown trout and sculpin kept up to 3 weeks after tagging. The laboratory test resulted in 100% survival and tag retention. In the field, all sculpin >70 mm and brown trout >110 mm (total length) were PIT‐tagged in the October electrofishing campaign. We used 12‐mm HDX – half‐duplex – tags (TRPGR30TGC; length 12.0 mm; diameter 2.1 mm; weight in the air 0.1 g; Texas Instruments, Dallas, TX) for sculpin (*n *=* *182) and juvenile trout of 110–150 mm length (*n *=* *50). Adult trout >150 mm (*n *=* *161) were tagged with 23‐mm HDX tags (RI‐TRP‐RR3P; 23.1 mm; 3.85 mm; 0.6 g; Texas Instruments). Tags were surgically inserted into the body cavity through a small incision of 3 mm width for the 12‐mm tags and 6 mm width for the 23‐mm tags. For the sculpin, the incision was made about 2 mm off the mid‐ventral line, between the pelvic girdle and the anus (Knaepkens et al. 2007). For trout, the incision was placed at the posterior tip of the pectoral fin 1–5 mm off the mid‐ventral line (PIT Tag Steering Committee 1999). No sutures were used in order to not further extend the tagging procedure. Tag/body mass ratio averaged 1.7% for the sculpin (0.7–3.1%), 0.5% for the juvenile trout (range 0.3–0.7%), and 1.3% for the adult trout (0.2–2.1%). All fish were released along the fished subreach after full recovery after two hours. No mortalities occurred.

### Fish tracking

Tracking for fish was performed on foot at 7‐day intervals during daylight hours from early November through early May 2011 (26 trackings in total) using a mobile radio frequency identification system with a 90‐cm ring antenna mounted on a 2.5‐m pole (Leonie System, Technologie Aquartis SENC, Québec, Canada). Reader and 12‐V DC rechargeable battery were enclosed in a backpack; the palmtop computer was mounted on the field map. Precedent laboratory tests in air following Linnansaari et al. (2007) revealed detection ranges of 85 cm for the 23‐mm tags and 45 cm for the 12‐mm tags if the longitudinal tag axis was placed parallel to the antenna plane and 105 cm and 60 cm, respectively, for perpendicular orientation of the tag. Detection ranges in the field were observed to be slightly higher, as identified at each tracking occasion for four stationary instream tags (“test tags”). The higher detection ranges in the field are probably due to the low degree of electromagnetic disturbance (Technologie Aquartis SENC, personal communication). We were able to pass the antenna over the entire width of the stream during both ice‐free and iced‐over conditions. To reduce any tracking‐induced disturbance of fish or ice, tracking was performed from the stream margins in early and late winter, with the ring antenna floating on the water by means of a bicycle tube. In the presence of a solid layer of surface ice, we tracked walking on the ice. Excessive snow was removed three times after heavy snowfall (>25 cm; December 21, January 11, March 13) to prevent conflicts with the limited detection range of the antenna. Fish positions were determined with a 0.5‐m accuracy using the blind spot method described in Linnansaari et al. (2007). Positions were plotted on the detailed field map, using a coordinate system with 1‐m grid cells and the distances to next reference trees. We worked in a two‐person team with fix duties (tracking, mapping) in order to guarantee a standard procedure.

### Data processing

Fish locations were transferred into the electronic maps in ArcMap for data processing. For each fish, we built up a detection history covering the 26 tracking events, with detections within the entire study reach coded as 1 and missing detections coded as 0. Following Pradel et al. (1997), we defined transients as individuals that are tagged, “released, and which then permanently emigrate from the sample, such that they are no longer available for detection in the future”. In order to avoid misinterpretation with isolated tags that were either lost by the fish after tagging or remained in the channel after the fish had died, we treated those fish as transients also who had shown no activity at all or only linear downstream movement. All other fish were treated as residents. Within‐site mortality during the study was assessed from a prolonged cessation of movements (Linnansaari and Cunjak 2010) and was confirmed in the spring electrofishing. The detection histories of the respective fish were set to zero as from the last tracking occasion with discernible upstream movement (Linnansaari et al. 2009), that is, changes in position beyond tracking accuracy of 0.5 m.

### Analysis

The detection histories were analyzed by means of the software MARK that uses numerical maximum likelihood techniques (Cormack‐Jolly‐Seber models; CJS models) to estimate apparent survival, *φ*, and detection probability, *p*, in mark–recapture data (White and Burnham 1999). Biological, environmental, and methodological factors were considered in the modeling, either as categorical grouping variables, as continuous individual covariates, or as temporal constraints. The three groups of fish (sculpin, juvenile brown trout, and adult brown trout) were treated as grouping variables. Apart from accounting for effects induced by tag size, this variable allowed for the indication of species‐specific differences, given that group‐specific model parameters (e.g., slope coefficients/effect sizes) are calculated. Three biometric characteristics measured at the tagging in autumn were applied as individual covariates to account for physical heterogeneity among individuals (body length [mm]; weight [g]; Fulton's condition index, Ricker (1975)). Another three covariates were applied to characterize the range of habitats and positions used by each individual fish over the entire study period – the median water depth in the subreach [cm], the mean distance to the closest end of the study reach [m] and the variance of the maximal depths in the subreach as a measure of structural complexity [coefficient of variation, CV; Jungwirth et al. 1995]). The approximated average ice thickness [cm] was included as a linear temporal constraint limiting the detection of tags due to the restricted detection range of the antenna.

We started the analysis with a fully time‐dependent Cormack–Jolly–Seber model {*φ*(g × t)*p*(g × t)} and 27 sampling occasions (initial marking and 26 trackings). In the starting model, t represents time and g stands for the grouping variable, that is, the three groups of fish. Transients were excluded from the analysis as classical CJS models assume that all individuals within a group have the same probability of subsequent detection (Cooch and White 2011). Data sparseness resulting from the large number of different detection histories led to a highly asymmetrical deviance residual plot. We therefore pooled trackings from two adjacent weeks into a single occasion, that is, a fish detected in one or both weeks was coded as 1, whereas a missing detection in both weeks was coded as 0. Doing so, we ended up with individual detection histories comprising 14 occasions. The overall detection pattern was not influenced by this pooling. The GOF (goodness‐of‐fit) of the fully time‐dependent model was tested by means of the median c‐hat procedure (Cooch and White 2011). Individual covariates were excluded from this step in the analysis (Cooch and White 2011). The resultant GOF‐measure ĉ (“c‐hat”) was integrated in MARK to adjust the fully time‐dependent model including individual covariates as well as all follow‐up models for a potential lack of fit due by overdispersion. In a next step, we modeled apparent survival and detection probability by including various combinations of factors described above (groups, covariates, and linear temporal constraint), that is, we did not test all possible models in order to avoid spurious results (Anderson et al. 2001). From the resulting list of candidate models, we selected the most parsimonious model by means of the corrected Akaike's information criterion (QAIC_c_), that is, the model which explained most of the variation in the data, while using the fewest model parameters. This is the model with the lowest QAIC_c_ value. From this top‐ranked model, estimates of apparent survival and detection probability were obtained. All models were run using the logit link function, and simulated annealing as an alternative optimization algorithm. A potential effect of the degree of maturity (spawning vs. spent) on the detection history of adult trout (transient vs. residents) was analyzed by means of the Mantel‐Haenszel chi‐squared test (Linnansaari et al. 2009).

## Results

### Subreach characteristics and ice thickness

Stream width and depth in autumn averaged 3.13 m and 0.21 m, respectively, with quite some variability across the 11 subreaches (Table 1). Structural complexity as expressed by the CV of the maximum depth ranged from 19.2 in subreach 10 to 46.1 in subreach 2. We caught 46.7–137.8 sculpin 100 m^−1^ and 28.5–90.4 trout 100 m^−1^. The number of tagged fish ranged from 27 to 52 per subreach.

**Table 1:** Description of the eleven subreaches studied in Smörbäcken. Environmental and biological parameters were measured in autumn 2010. The standard deviations for length and width are given in parentheses

Subreach	1	2	3	4	5	6	7	8	9	10	11
Length (m)	42	49	58	47	38	58	48	56	48	48	56
Mean wetted width (m)	3.79 (0.89)	3.51 (0.86)	3.34 (0.62)	3.09 (0.60)	4.12 (1.50)	3.55 (0.74)	3.13 (0.99)	2.67 (0.54)	3.00 (0.63)	2.20 (0.35)	2.56 (0.66)
Mean depth (cm)	21.0 (12.3)	23.7 (13.5)	23.7 (11.6)	21.7 (9.2)	20.1 (10.8)	21.9 (10.8)	21.1 (9.2)	25.8 (12.3)	16.6 (7.4)	19.1 (9.7)	17.3 (12.1)
Structural complexity (–)	40.0	46.1	40.8	28.8	36.8	32.5	24.2	31.5	23.1	19.2	39.1
Sculpin density (ind. 100 m^−1^)	109.6	63.7	53.7	51.1	137.8	91.4	99.3	53.5	83.1	68.2	46.7
Trout density (ind. 100 m^−1^)	88.2	90.4	55.4	49.0	59.8	55.2	49.7	28.5	35.3	35.2	75.4
Number of fish tagged	39	52	46	29	33	39	35	28	34	27	31
Sculpin	17	17	16	7	15	19	18	10	22	22	19
Juvenile trout	5	13	8	3	5	0	3	2	0	2	9
Adult trout	17	22	22	19	13	20	14	16	12	3	3

Ice formation started in early November, with different types of ice forming. Continuous growth of the ice toward the substratum and on the surface by repeated aufeis events, and minor snow accumulation despite the three shoveling occasions resulted in an average measured thickness of the snow–ice pack of 34.0 cm by the end of January. Ice thickness was variable across the study reach, ranging from 16 cm to 84 cm by the end of January (CV = 0.33). Average measured ice thickness closely compared to the approximated values following the formula by Ashton (1986) and Lundberg and Feiccabrino (2009), which was 34.5 cm for late January (Fig. 3). Approximated ice growth was most pronounced between late November and early December when supercooling of the stream water was measured. Ice growth slowed down toward late winter, with a maximum thickness of 46.4 cm reached before breakup in mid‐April.

### Return rate

**Figure 1:**
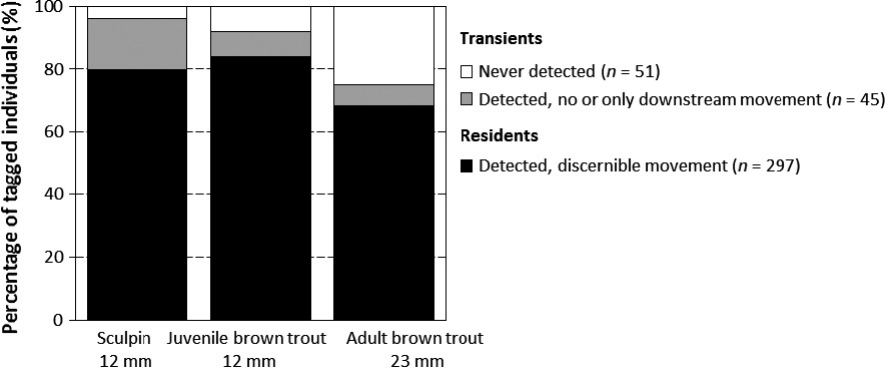
Percentage of individuals with different detection histories. Millimeter values refer to tag size. The number of individuals per detection history is given in brackets.

Over the entire study period, 297 individuals or 76% of all tagged fish showed discernible upstream or lateral movement, that is, they were treated as residents (Fig. 1): 145 sculpin (49% of all residents and 80% of all tagged sculpin), 42 juvenile brown trout (14%; 84%), and 110 adult brown trout (37%; 68%). Four percent of all sculpin (seven individuals), 8% of the juvenile, and 25% of the adult trout (four and 40 individuals, respectively) were never detected within our study reach, that is, they were transients. Thirty sculpin (17%), four juvenile, and 11 adult trout (8% and 7%, respectively) showed no or only linear downstream movement and were not recaptured at the electrofishing in spring, so they were also treated as transients. Tag loss was observed for nine adult brown trout at the electrofishing in spring (incision, but no tag). For five of these trout, the tag number could be explicitly identified based on their characteristic length; two of these tags had never been detected and three had been classified as “no or only downstream movement.” There was a significant relationship between the degree of maturity and the detection history (chi‐square = 8.69; *P *<* *0.01). The probability of spawning trout to become transients was 2.1 times higher than the one for the spent trout.

**Figure 2:**
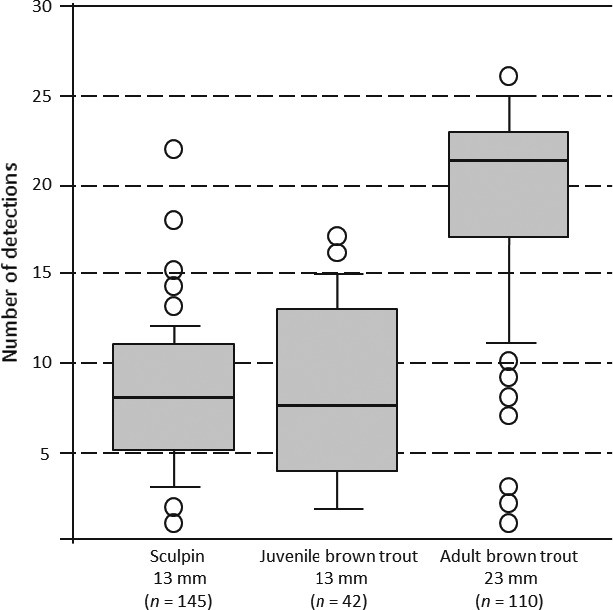
Distribution of the total number of detections among the three groups. Only the 297 residents are shown, that is, the individuals with discernible movement (see also Fig. 1). Millimeter values refer to tag size. The maximum number of detections possible was 26.

For the 297 residents, we collected 3615 positions over the 26 weekly trackings, whereof 1183 or 33% belonged to sculpin, 341 or 9% to juvenile trout, and 2091 positions or 58% to adult trout. Among the individuals detected, the total number of detections varied between groups, with the smaller individuals tagged with 12‐mm tags being less frequently detected (Fig. 2). A given sculpin was on average detected in 31% of the trackings (i.e., eight detections; median value), and a similar value was found for the juvenile trout (7.5 detections; 29%). Conversely, an adult trout was detected in 81% of the trackings on average (i.e., 21 detections).

The return rate varied between sampling occasions and groups (data not shown). More than 80% of all resident sculpin could be found at the early winter trackings, before the return rate dropped below 5% in late February. From early April on, a sharp increase in the return rate was observed with a return rate of 71% at the last tracking, leading to a total variation in return rate by factor 21.0 (CV = 0.80). A similar, but less pronounced pattern was found for the juvenile brown trout for which the return rate varied by factor 11.3 (CV = 0.59). Conversely, the return rate for the adult brown remained at a constantly high level of ≥70% and showed little temporal variation (factor 1.4; CV = 0.11).

### Model outcome

Our data were slightly overdispersed as illustrated by a ĉ of 1.21 (SE = 0.01) resulting from the GOF‐testing of the fully time‐dependent model. The default value of 1 was therefore adjusted in MARK. Two of the 39 candidate models were within 20 ΔQAIC_c_ units from the top‐ranked model – the one with the lowest QAICc value, {*φ*(t × cond)*p*(g × ice + complex × t)} (Table 2). Because the top‐ranked model carried 69.5% of QAICc weight (Table 2), it was chosen to obtain estimates of apparent survival and detection probability.

**Table 2:** The top three models for estimating apparent survival, *φ*, and detection probability, *p*, for sculpin, juvenile, and adult brown trout in Smörbäcken. ΔQAIC_c_ is the difference between the QAIC_c_ values of a given model and the top model. QAIC_c_ weight *w*_*i*_ reflects the relative support of a given model in the set of candidate models. The model likelihood illustrates the strength of evidence for this model and corresponds to the ratio in QAIC_c_ weights between the present model and the best model. In the last column, the number of estimable parameters is given. QAIC_c_ for the top model was 2652.60

Rank	Model	ΔQAIC_c_	QAIC_c_ weight *w*_*i*_	Model likelihood	Number of parameters
1	{*φ*(t × cond)*p*(g × ice + complex × t)}	0.00	0.6953	1.0000	29
2	{*φ*(t × cond)*p*(g × ice + complex × distance × t)}	1.68	0.3007	0.4325	43
3	{*φ*(t × cond)*p*(g × ice + depth × distance × t)}	10.34	0.0040	0.0057	43

Effects are abbreviated as follows: t = time; cond = physical condition; g = group (adult trout, juvenile trout, and sculpin); ice = approximated average ice thickness; complex = variance of the maximal depth in the subreach; distance = mean distance to closest study reach end; depth = mean water depth.

**Table 3:** Estimated apparent survival calculated from the top‐ranked model for all residents (no group effects). Apparent survival for time intervals 1 and 13 are not shown because they were not estimable in this model for the last sampling event (Cooch and White 2011)

Time interval	Apparent survival estimate	SE
2	0.999	0.001
3	0.999	0.001
4	1.000	0.001
5	1.000	0.000
6	0.999	0.001
7	0.995	0.002
8	0.998	0.001
9	1.000	0.000
10	0.997	0.002
11	0.999	0.001
12	0.999	0.001

#### Detection probability

**Figure 3:**
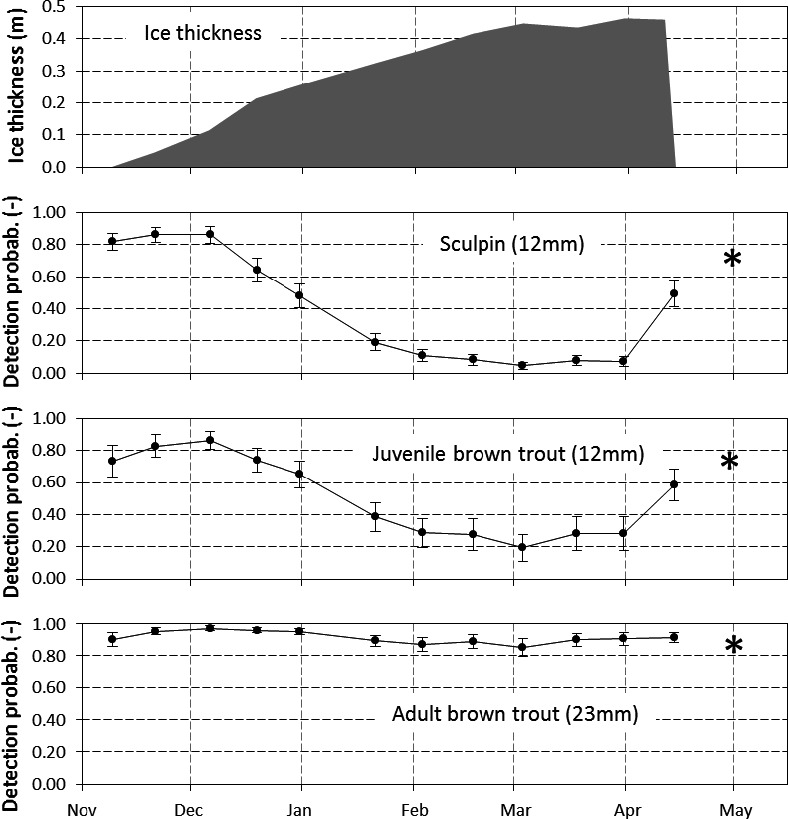
Temporal variation in the ice thickness and in the estimated detection probability for the three groups of fish. Tag size is given in brackets. (*) Detection probabilities for time interval 13 are not shown because they were not estimable in this model for the last time interval (Cooch and White 2011).

**Figure 4:**
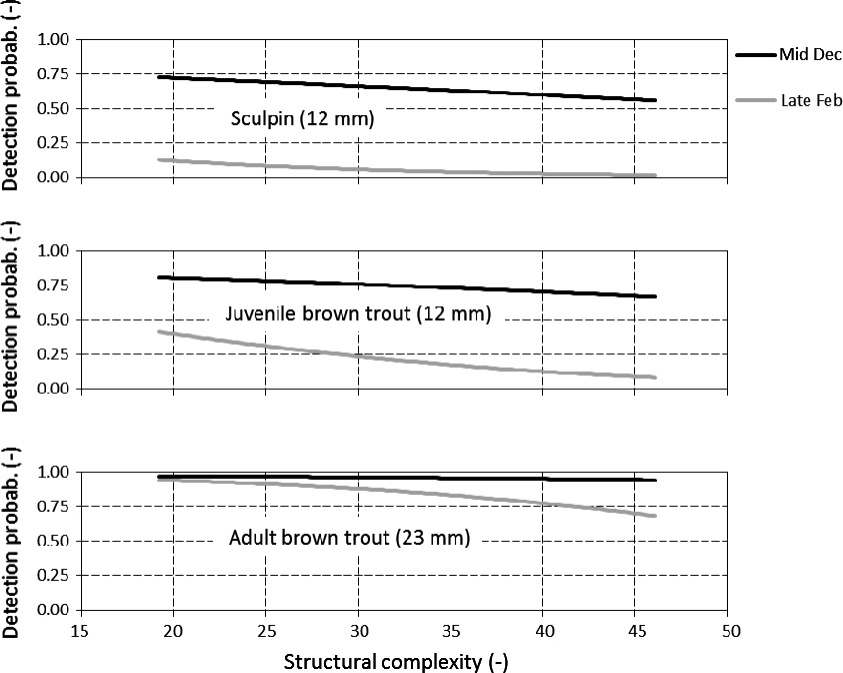
Estimated detection probability as a function of structural complexity illustrated for two time intervals, mid‐December and late February, respectively. Effect size in mid‐December was −0.028 (95% CI = −0.046, −0.009) and −0.076 in late February (95% CI = −0.103, −0.049).

In the top‐ranked model, detection probability was driven by ice thickness (“ice”) which showed group‐specific effects, and the habitat parameter “structural complexity” (“complex”; in all three top‐ranked models) whose effect was time‐dependent. Detection probability was independent from the physical condition of the fish or any other biological variable. Biweekly detection probabilities as obtained from the top‐ranked model ranged from 0.05 to 0.86 for sculpin, from 0.20 to 0.86 for the juvenile trout, and from 0.85 to 0.97 for the adult trout (Fig. 3). Detection probability was negatively correlated with ice thickness for all three groups (Fig. 3), with the effect being largest for sculpin and juvenile brown trout. Structural complexity was negatively correlated with detection probability across all time intervals (Fig. 4). The effect was stronger in mid‐winter as compared to early or late winter (see effect sizes in Fig. 4).

#### Apparent survival

**Figure 5:**
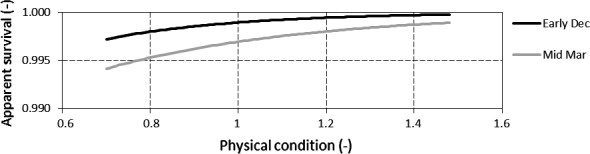
Estimated apparent survival as a function of individual physical condition illustrated for two time intervals, early December and mid‐March, respectively. Effect size in early December was 3.255 (95% CI = 1.081, 5.429) and 2.187 in mid‐March (95% CI = 0.116, 4,258).

In the top‐ranked model, apparent survival varied over time and as a function of the individual physical condition (“cond”). Habitat parameters had no effect on the apparent survival, nor did the grouping variable or the other biometric characteristics of the fish. Biweekly apparent survival as obtained from the top‐ranked model was generally high and varied between 0.995 and 0.999 (Table 3). The physical condition effect was positive, meaning that individuals with a higher physical condition showed a higher apparent survival (see effect sizes in Fig. 5).

## Discussion

Our results show that detection probability decreased with growing ice volume and was smallest in structurally complex reaches. This relationship held across all three groups considered, with the strongest effects in the smaller‐sized fish that were tagged with 12‐mm tags. Apparent survival was generally high and dependent on the physical condition of the fish in autumn.

One‐fourth of all adult trout, but less than 10% of the juvenile trout and sculpin were transients. These fish had been tagged and released all across the study reach, that is, individuals tagged at the study reach borders did not have a higher probability of permanently leaving the study area, for instance, during post‐tagging movements (Enders et al. 2007). Percentages of >20% of transient individuals have been reported from various mark–recapture studies with stream‐resident salmonids (Gowan et al. 1994), illustrating within‐population heterogeneity in mobility (resident vs. mobile fractions of the population). Within our study reach, we observed similar median winter home ranges for all three groups of fish, but the largest variation was indeed found for the adult trout (C. Weber, unpublished data). Apart from such differences in spatial exploitation, the higher percentage of transient adult trout may also be influenced by the timing and type of tagging. First, we tagged toward the end of the spawning season of the trout, that is, we might have caught some adult fish in their temporary spawning habitats as spawning migrations of a few to several kilometers are well documented for stream‐resident brown trout (Elliot 1994; Ovidio et al. 1998). An effect of the spawning state on transience seems probable as among the transient individuals there were disproportionally more spawning trout than spent individuals. Furthermore, the emigration must have happened early after tagging given the high detection probability of the adult trout, and the detection pattern for the juvenile trout differed completely. Second, we tagged before a major decline in water temperature which has been shown to initiate habitat shift in juvenile and adult salmonid fish (Heggenes et al. 1993). Third, a certain percentage of missing detections for the adult trout must be attributed to tag loss within a few days after tagging. A loss rate of 5% as observed in the present study is at the lower end of the range reported in the literature if no sutures are used (Siikavuopio et al. 2009). We assume that in our case, tag retention was reduced by the spawning activity (Gries and Letcher 2002) as no tag loss was observed in the juvenile trout and sculpin, neither in the spring electrofishing nor in precedent laboratory tests. Tag loss in the adult trout might have been reduced using sutures (Roussel et al. 2000) – an approach we neglected given the additional time needed for handling which can be stressful for fish, particularly at low temperatures.

There are two ways to prove the fate of transient individuals – installing stationary antennas at the study reach borders (Zydlewski et al. 2006) and tracking adjacent reaches (Stickler et al. 2008) – both of which we had to reject in the present study. Both swim‐through and swim‐over antennas are difficult to keep in place and to operate even in small streams when ice forms at the surface, in the water column and at the stream bottom (Stickler et al. 2008). Furthermore, powering the reading devices by line current was unrealistic given the remoteness of the study site. We did not conduct additional trackings, given the heavy snowpack and the high tracking effort.

One particular challenge in remote tracking of PIT‐tagged fish is to cope with lost or dead tags that may bias the analysis of movement patterns, detection probability, or apparent survival. To avoid misinterpretations, we excluded 14.7% of detected tags from the analysis due to their spatial pattern (no or only downstream movement). We believe this to be a reasonable, but rather restrictive approach as (1) at the spring electrofishing, we recaptured fish from all three groups with similar spatial patterns as the excluded tags, and (2) tag loss and post‐tagging mortality had been inexistent in precedent laboratory tests with sculpin and juvenile trout.

The detection probability varied between groups and over time as a function of increasing ice thickness. The surface ice layer defined the position of the antenna. Accordingly, the tracking from mid‐December on was difficult, particularly for the sculpin and the juvenile trout that were tagged with 12‐mm tags which have a maximum detection range of 0.5 m only. The detection probability for these two groups decreased considerably with increasing thickness of the ice layer, and detection was restricted to locations where the ice was relatively thin or the water depth relatively shallow. However, with the decay and breakup of the ice in spring, detection probability for the small tags rose again. The effect of ice thickness on detection probability was visible, but much less pronounced for the adult brown trout tagged with 23‐mm tags. The detection range for these larger tags is much bigger (>80 cm), enabling the effective scanning of most habitats in our rather shallow study reach.

Group‐specific differences in habitat use may have further increased the effect of the tag size and ice formation. Sculpin are bottom‐dwellers, which are reported to conceal themselves in the substratum during low‐flow conditions (Bless 1990, cited in Lucas and Baras 2001) as they prevail in boreal streams during winter. A similar behavior is also known for juvenile salmonids in partly frozen‐over systems (Heggenes and Saltveit 1990). Bottom‐dwelling or substratum‐concealing individuals easily fall outside the detection range when antenna maneuverability is limited by surface ice. In contrast, there is a higher chance to detect individuals exploiting the entire water column. Interestingly, the lowest detection probabilities (<10%) were exclusively reached by the sculpin, whereas for the juvenile trout detection probabilities never dropped below 20%. This could be an indication of that in completely frozen‐over systems such as in our study stream, juvenile trout are in less need of substrate concealing than in partly frozen streams where they hide from homeothermic mammalian and avian predators (Heggenes et al. 1993; Huusko et al. 2007).

Apart from technical limitations, ice formation can, in concert with decreasing water temperatures and lower activity, also positively affect detection probability, as illustrated by the rising detections of adult trout in early winter (Fig. 3). Adult trout were increasingly spotted in less structured habitats covered by surface ice. These habitats were either avoided under open‐water conditions, possibly due to too little cover (Linnansaari et al. 2009), or the tracking in open water resulted in a fright response (Cucherousset et al. 2005).

The subreaches in our study reach differed in structural complexity (undercut banks, woody debris, and substratum) which was reflected by differences in the variance of the maximal depth (Table 1; Jungwirth et al. 1995). Individuals in structurally complex, deeper subreaches were generally less frequently detected than those occurring in poorly structured subreaches. These findings corroborate observations by Linnansaari and Cunjak (2010) who could not track salmon parr under logjams in the presence of surface ice due to limited maneuverability of the antenna. Detection probability of slimy sculpin was inversely related to the percentage of boulders in five small New Brunswick streams (Keeler et al. 2007). A reduced detection probability in structurally complex, deep habitats, such as pools, leads to an underestimation of their importance as winter habitats (Stickler et al. 2008).

Traditional approaches to estimate winter mortality have compared fish densities in autumn with those in spring (e.g., Bradford et al. 2001; Lund et al. 2003). With remote detection, temporal dynamics in apparent survival and the factors that govern these dynamics can be determined (Hewitt et al. 2010). A major interest is linking demographic processes with spatial dynamics, such as habitat‐specific estimates of demographic processes (Lowe et al. 2006). In our study, system apparent survival, which was generally high, was not directly affected by the environmental parameters studied, and also group effects proved to be irrelevant. Rather, an individual physical characteristic such as the body condition in autumn positively influenced apparent survival. Body condition reflects the fish's physical reserves which have been shown to decline over winter as a function of the energetic demands (Cunjak 1988). Body condition has been proved to be a better predictor than body size who did not prove to be related to over winter survival in three Norwegian streams (Lund et al. 2003).

Our study has broad relevance in that it demonstrates that remote tracking of PIT‐tagged fish in frozen‐over streams is feasible, even when using the recently developed 12‐mm HDX tags for smaller‐sized individuals. This enables the study of different species and age classes with clearly differing ecological requirements and responses to winter dynamics, such as the bottom‐dwelling sculpin and water‐column species such as the brown trout. However, prerequisites for a successful tracking are that the study streams are shallow (e.g., mean water depth <30 cm, depending on the expected ice volume) and that frequent tracking is possible (e.g., snow depth, accessibility).

Furthermore*,* a critical determination of factors affecting return rate and the underlying detection and survival probabilities is needed for remote PIT‐tracking (Zydlewski et al. 2006). This procedure is comparable to the calculation of capture probabilities in quantitative electrofishing which may also show spatiotemporal variability depending on water temperature or cover availability (White et al. 1982). Without a consideration of driving factors, tracking results are difficult to interpret, and findings from different study sites, years, species or age classes cannot be directly compared. A good knowledge of the study site at the time of the tracking (e.g., water depth, ice thickness) is required to determine the area that is actually scanned by the antenna (Linnansaari et al. 2007). Furthermore, it is crucial to include characteristics of the individual fish that may affect apparent survival or detection probability, such as the physical condition or the size and position of the home range relative to the study reach length and borders, respectively.

## Conflict of Interest

None declared.
